# Monoallelic maternal expression of *STAT5A *affects embryonic survival in cattle

**DOI:** 10.1186/1471-2156-10-13

**Published:** 2009-03-10

**Authors:** Hasan Khatib, Christian Maltecca, Ricky L Monson, Valerie Schutzkus, Jack J Rutledge

**Affiliations:** 1 Department of Dairy Science, University of Wisconsin-Madison, Madison, WI 53706, USA; 2 Department of Animal Sciences, University of Wisconsin-Madison, Madison, WI 53706, USA; 3 Department of Animal Science, North Carolina State University, Raleigh, NC 27695, USA

## Abstract

**Background:**

Reproductive disorders and infertility are surprisingly common in the human population as well as in other species. The decrease in fertility is a major cause of cow culling and economic loss in the dairy herd. The conception rate has been declining for the past 30–50 years. Conception rate is the product of fertilization and embryonic survival rates. In a previous study, we have identified associations of several single nucleotide polymorphisms (SNPs) in the signal transducer and activator 5A (*STAT5A*) with fertilization and survival rates in an *in *vitro experimental system. The objectives of this study are to fine map the *STAT5A *region in a search for causative mutations and to investigate the parent of origin expression of this gene.

**Results:**

We have performed a total of 5,222 fertilizations and produced a total of 3,696 in vitro fertilized embryos using gametes from 440 cows and eight bulls. A total of 37 SNPs were developed in a 63.4-kb region of genomic sequence that includes *STAT5A*, *STAT3*, and upstream and downstream sequences of these genes. SNP153137 (G/C) in exon 8 of *STAT5A *was associated with a significant variability in embryonic survival and fertilization rate compared to all other examined SNPs. Expression analysis revealed that *STAT5A *is primarily monoallelically expressed in early embryonic stages but biallelically expressed in later fetal stages. Furthermore, the occurrence of monoallelic maternal expression of *STAT5A *was significantly higher in blastocysts, while paternal expression was more frequent in degenerative embryos.

**Conclusion:**

Our results imply that *STAT5A *affects embryonic survival in a manner influenced by developmental stage and allele parent of origin.

## Background

The intense genetic selection for milk production traits in dairy cattle over the past 50 years, which has resulted in a tremendous increase in productivity, has been coupled with a significant decrease in fertility. Indeed, reproductive performance in dairy cattle is now clearly suboptimal as revealed by the sharp reduction in first-service pregnancy rates from 70% to 40% [1-3]. Although there are substantial genetic effects that contribute to this infertility, little progress has been made on the identification of major genes affecting reproduction traits [4].

The signal transducer and activator (STAT) proteins are transcription factors that are known to play an important role in cytokine signaling pathways as signal transducers in the cytoplasm and as transcription activators in the nucleus [5]. In a previous study, using the candidate pathway approach, *STAT5A *was chosen as a candidate gene for early embryonic survival because of its roles in embryonic development and in the signal transduction pathway of interferon-tau (IFNT), which has a key role in the initiation and maintenance of pregnancy in ruminants [6]. We identified 12 single nucleotide polymorphisms (SNPs) and found that some of them were associated with fertilization and survival rates in a population of 1,500 in vitro fertilized (IVF) embryos produced from three sires and 160 dams [7]. However, it was not clear whether the observed effects were related to SNPs in *STAT5A *or to other SNPs in linkage disequilibrium with a causative mutation in the *STAT5A *region. Thus, one objective of this study was to fine map the *STAT5A *region by identification of new SNPs. Furthermore, recent studies have shown that *STAT5A *is expressed in oocytes at the metaphase II stage (before fertilization) and in 2-cell, 4-cell, morula, and blastocyst stages [8] which suggests a possible role of this gene in fertilization and early embryonic development. Thus, to better understand the mechanisms by which *STAT5A *affects fertilization and embryo survival, the second objective of this study was to investigate the expression patterns of this gene in blastocysts and degenerative embryos and to analyze its sequence characteristics.

## Results

### Polymorphism identification and association of STAT5A with embryonic survival and fertilization rate

We have constructed a unique resource population of IVF embryos with the aim of identifying genes affecting fertility traits in cattle. A total of 5,222 fertilizations were performed in vitro using oocytes from 440 ovaries and semen from eight sires which resulted in a total of 3,696 embryos.

In a previous study we identified 12 SNPs in *STAT5A *and found association of some of these SNPs with early embryonic survival and fertilization rate [7]. In this study, we extended our SNP search to include upstream and downstream sequences of *STAT5A *and *STAT3 *– about 3.5 kb apart – and in exons of *STAT3*. Using the pooled DNA sequencing approach, a total of 25 new SNPs were identified and confirmed in individual sequencing of 10–15 DNA samples from the pools (Table 1). Overall, a total of 37 SNPs over more than 63 kb genomic region were employed in association tests with fertility traits. Of those 37 SNPS, 14 were located upstream of *STAT5A*, 12 were in *STAT5A*, two were in *STAT3*, and nine were upstream of *STAT3 *(Additional file 1).

**Table 1:** Primer sequences and amplification product sizes.

**Primer**	**Sequence**	**Product size (bp)**	**SNP (nucleotide number)^1^**
5PSTAT5-3F 5PSTAT5-4R	CTGTAGTTGTCCCTGCAGAAG CTGTGTCAGCCTCACCCTCTC	840	134357

5PSTAT5-5F 5PSTAT5-6R	GAGAGGGTGAGGCTGACACAG CTGAGGCATGCAGACTCTTAG	921	134828; 134920; 135162; 135249; 135397

5PSTAT5-11F 5PSTAT5-12R	CACGGAGATACTTCCTGGAAGG TGAACCGTGGCACACTCGTG	840	137887; 138012

5PSTAT5-13F 5PSTAT5-14R	CACGAGTGTGCCACGGTTCAG CCAAACATCTGGCTGGGTTG	940	138242; 138299; 138337; 137338; 138596; 138653

SPSTAT3-21F 5PSTAT3-21R	CTTAAGAACTGGGGTTCCCGG CTGCTCTCCTGAATATATGC	681	197390; 197429; 197456; 197558; 197602; 197608; 197713; 197718; 197740

STAT3F8 STAT3R8	TCCCCCAAGGATCCTACG CCATAGTACCAGACAACTGG	420	171005

STAT3F12 STAT3R12	CACCCCCTGCATTGGAAGCG CTTCTACTTGAGCATGTACAGGG	653	177338

STAT3F12A STAT3R12A	CTCTCCTGCTCAGCTACATC GGACTTTCAAAGAGACTCGG	469	177338

STATF1 STATR1	GAGAAGTTGGCGGAGATTATC CCGTGTGTCCTCATCACCTG	820	153137

STAT14 STAT13	GAGGAGATGCTGGCTGAGGT TTCAGGGGACAGGACTCTGG	440	153137

STAT14 (exon8) STAT11 (exon 11)	GAGGAGATGCTGGCTGAGGT CCGGTCAGCTCGCTTGATCC	360	153137

STAT3-1 (exon 13) STAT3-2 (exon 9)	ACCGCATCTCTGCTCTCTCAG GCCATTAGTCATCAAGACCG	253	177338

b-actin F b-actin R	CAGCACAATGAAGATCAAGATCATC AAAGGGTGTAACGCAGCTAACAGT	191	

^1^According to GenBank accession number NW_001493680

**Figure 1:**
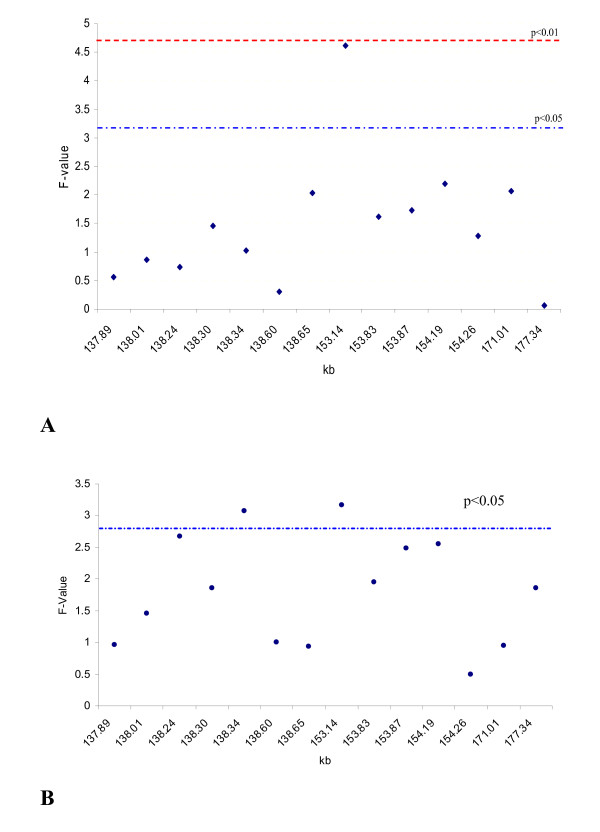
**Association analysis of 14 SNPs with (A) survival rate and (B) fertilization rate with a population of gametes from 440 ovaries and eight bulls**. A total of 5,222 fertilization and 3,696 embryos were used to collect phenotypic records of survival and fertilization rates. SNP153137 in exon 8 of STAT5A showed the highest significant effect on embryonic survival (A) and fertilization rate (B). The SNPs in numeric order were 137,887; 138,012; 138,242; 138,299; 138,337; 138,596; 138,653 (in upstream sequences of *STAT5A*); 153,137; 153,827; 153,866; 154,186; 154,261 (in *STAT5A*); 171,005; and 177,338 (in *STAT3*). Significance threshold for the association determined via permutation with 250 iterations.

**Figure 2:**
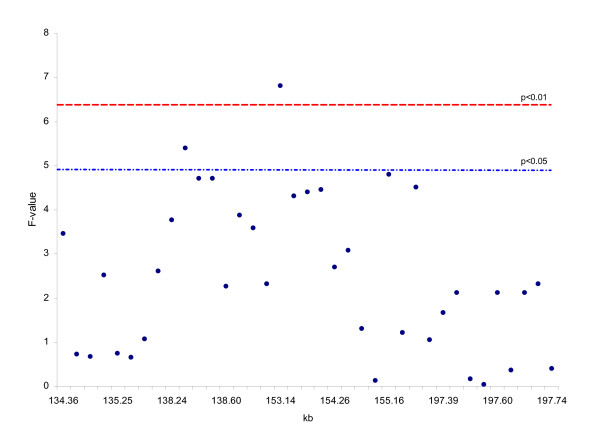
**Association analysis of 37 SNPs in selective genotyping of high and low embryo survival rate groups**. SNP153137 in exon 8 of STAT5A showed the highest significant effect on early embryonic survival. Significance thresholds for the association determined via permutation with 250 iterations.

First, the whole "ovary" population (n = 440) was genotyped for 14 SNPs – seven upstream of *STAT5A*, five within *STAT5A*, and two within STAT3 – and analyzed for embryonic survival and fertilization rate. The SNP153137 (G/C) in exon 8 of *STAT5A *(SNP12195 in our previous study) was associated with the highest significant effect (*P *= 0.0105) for survival rate (Figure 1A). The survival rate of embryos produced from CC dams was 12.8% higher than embryos produced from GG dams (*P *= 0.0027) (Table 2). Figure 1B shows that SNP153137 and SNP138337 (2.6 kb upstream of *STAT5A*) had the highest significant associations (*P *= 0.0466 and *P *= 0.0482, respectively) with fertilization rate. For SNP153137, the fertilization rate of oocytes obtained from CC dams was 7.7% higher than those from GG dams (*P *= 0.0258) (Table 2). For SNP138337, the fertilization rate of oocytes from AA dams was 5.7% lower than AG dams (*P *= 0.0213). Second, to fine-map the *STAT5A *region, we genotyped the other 23 SNPs identified in *STAT5A*, *STAT3*, and in their upstream and downstream sequences in the 100 dams with the highest embryonic survival rate and the 84 dams with the lowest survival rate. SNP153137 again showed the highest significant effect (*P *= 0.0014) on survival rate (Figure 2). The estimate of the effect of CC dams was 52.7% survival rate vs. 25.9% for GG dams.

**Table 2:** Contrasts (standard errors ±) for survival and fertilization rates analyzed for 14 SNP upstream of *STAT5A*, in *STAT5A*, and in *STAT*.

SNP/Genotypes	Difference in survival rate	P value	Differenc in fertilization rate	P value
SNP137887 (upstream STAT5A)				

AA vs. AG	-0.029 ± 0.030	0.3429	-0.035 ± 0.025	0.1715

AA vs. GG	0.009 ± 0.053	0.8552	-0.024 ± 0.044	0.5929

AG vs. GG	0.039 ± 0.053	0.4674	0.011 ± 0.045	0.7965

SNP138012 (upstream STAT5A)				

GG vs. GT	-0.003 ± 0.032	0.9234	-0.044 ± 0.027	0.0990

GG vs. TT	-0.056 ± 0.045	0.2150	-0.038 ± 0.038	0.3113

GT vs. TT	-0.053 ± 0.044	0.2297	0.005 ± 0.037	0.8739

SNP138242 (upstream STAT5A)				

CC vs. CT	-0.027 ± 0.031	0.3798	-0.054 ± 0.024	0.0291

CC vs. TT	-0.051 ± 0.048	0.2824	-0.053 ± 0.038	0.1654

CT vs. TT	-0.024 ± 0.048	0.6150	0.001 ± 0.038	0.9709

SNP138299 (upstream STAT5A)				

AA vs. AG	0.055 ± 0.039	0.1591	0.037 ± 0.031	0.2429

AA vs. GG	0.070 ± 0.042	0.0975	0.062 ± 0.033	0.0678

AG vs. GG	0.014 ± 0.033	0.6640	0.025 ± 0.026	0.3458

SNP138337 (upstream STAT5A)				

AA vs. AG	-0.034 ± 0.031	0.2655	-0.057 ± 0.024	0.0213

AA vs. GG	-0.058 ± 0.047	0.2213	-0.060 ± 0.037	0.1091

AG vs. GG	-0.023 ± 0.047	0.6217	-0.003 ± 0.037	0.9328

SNP138596 (upstream STAT5A)				

CC vs. GC	-0.187 ± 0.250	0.4559	-0.204 ± 0.199	0.3085

CC vs. GG	-0.1767 ± 0.248	0.4778	-0.172 ± 0.198	0.3868

GC vs. GG	0.010 ± 0.036	0.7822	0.031 ± 0.029	0.2788

SNP138653 (upstream STAT5A)				

CC vs. GC	0.077 ± 0.040	0.0586	0.036 ± 0.032	0.2525

CC vs. GG	0.079 ± 0.044	0.0716	0.046 ± 0.034	0.1833

GC vs. GG	0.002 ± 0.033	0.9490	0.009 ± 0.026	0.7155

SNP153137 (STAT5A)				

CC vs. GC	0.067 ± 0.036	0.0682	0.021 ± 0.029	0.4771

CC vs. GG	0.128 ± 0.042	0.0027	0.077 ± 0.034	0.0258

GC vs. GG	0.061 ± 0.032	0.060	0.056 ± 0.026	0.035

SNP153827 (STAT5A)				

AA vs. AC	0.008 ± 0.037	0.8243	-0.036 ± 0.029	0.2260

AA vs. CC	-0.069 ± 0.048	0.1501	-0.072 ± 0.037	0.0521

AC vs. CC	-0.078 ± 0.044	0.0792	-0.036 ± 0.034	0.2876

SNP153866 (STAT5A)				

CC vs. CT	0.075 ± 0.042	0.0809	0.058 ± 0.032	0.0744

CC vs. TT	0.078 ± 0.047	0.0996	0.079 ± 0.036	0.0292

CT vs. TT	0.003 ± 0.036	0.9241	0.020 ± 0.027	0.4607

SNP154186 (STAT5A)				

AA vs. AG	-0.022 ± 0.035	0.5232	-0.028 ± 0.027	0.3007

AA vs. GG	-0.092 ± 0.045	0.0417	-0.078 ± 0.031	0.0250

AG vs. GG	-0.069 ± 0.040	0.0896	-0.049 ± 0.031	0.1160

SNP154261 (STAT5A)				

AA vs. AG	0.165 ± 0.103	0.1113	0.078 ± 0.080	0.3266

AA vs. GG	0.140 ± 0.097	0.1504	0.063 ± 0.075	0.4065

AG vs. GG	-0.024 ± 0.042	0.5579	-0.016 ± 0.032	0.6231

SNP171005 (STAT3)				

GG vs. GT	0.009 ± 0.039	0.8024	0.023 ± 0.030	0.4411

GG vs. TT	0.061 ± 0.036	0.0877	0.039 ± 0.028	0.1696

GT vs. TT	0.051 ± 0.032	0.1069	0.015 ± 0.025	0.5467

SNP177338 (STAT3)				

CC vs. CT	-0.011 ± 0.040	0.7804	-0.001 ± 0.031	0.9891

CC vs. TT	-0.015 ± 0.044	0.7231	0.05 ± 0.034	0.1408

CT vs. TT	-0.004 ± 0.036	0.8981	0.051 ± 0.028	0.0682

### Expression Analysis of STAT5A and STAT3

The significant association of SNP153137 with survival rate prompted us to investigate the expression pattern of *STAT5A *in embryos at the blastocyst stage, in degenerative embryos, and in fetuses at different developmental stages. This SNP was used to assess the monoallelic vs. biallelic expression pattern of *STAT5A *in RT-PCR products amplified from blastocysts and degenerative embryos heterozygous for the SNP (Table 3). Genotyping of more than 300 embryos at Day 7 of development revealed 111 heterozygous embryos. Monoallelic expression of *STAT5A *differed significantly in degenerative embryos (96.4%) vs. blastocysts (78.3%). Furthermore, where *STAT5A *expression was monoallelic, it was maternally expressed (imprinted) in 87.1% of the blastocysts vs. 59.1% in the degenerative embryos. Paternal expression was found to be higher in degenerative (40.9%) compared to blastocysts (12.9%).

**Table 3:** Monoallelic and biallelic expression and parent-of-origin expression of *STAT5A *in heterozygous blastocysts and degenerative embryos for SNP153137.

Expression pattern	blastocysts	degenerative embryos
Biallelic expression	18/83 (21.7%)	1/28 (3.6%)

Monoallelic expression	65/83 (78.3%)*	27/28 (96.4%)

Known parent-of-origin	31	22

Maternal	27/31 (87.1%)	13/22 (59.1%)

Paternal	4/31 (12.9%)**	9/22 (40.9%)

* P = 0.047 Pearson's Chi-squared test with simulated p-value (Chi-squared test with continuity correction p = 0.050)

** P = 0.026 Pearson's Chi-squared test with simulated p-value (Chi-squared test with continuity correction p = 0.047)

To test whether monoallelic expression of *STAT5A *is developmental-age specific, the expression pattern was examined in a wide range of organs obtained from bovine fetuses at 68 to 90 days of age. The *STAT5A *gene was found to be biallelically expressed in all examined organs from all five heterozygous fetuses (see Materials and Methods). For *STAT3*, genotyping of 48 embryos for SNP177338 in exon 12 revealed 18 heterozygous embryos. Expression analysis showed that *STAT3 *was exclusively biallelically expressed.

## Discussion

### The Candidate Pathway Strategy for Choosing STAT5A as a Candidate Gene

The discovery of survival genes is a challenging task in all species because of the complex nature of this trait, the lack of phenotypic data, and the difficulties in choosing suitable candidate genes among many other reasons. Candidate genes are mostly chosen based on previous linkage mapping studies (positional candidate gene approach) and on comparative biological or physiological functions in other species [10]. In a previous study, *STAT5A *was chosen as a candidate gene for embryonic survival based on a candidate pathway rather than position or comparative function of the candidate gene [7]. In the candidate pathway approach, genes are chosen based on their biological functions in the metabolic pathway. When one gene of a pathway affects our target traits, other genes of the same pathway are likely to do so as well. Using the candidate pathway approach, we have shown that the fibroblast growth factor 2 (*FGF2*), also a member of the IFNT signal transduction pathway, is associated with embryonic survival in cattle [11].

### SNP identification and association of STAT5A region with embryonic survival and fertilization rate

The single SNP analysis revealed that SNP153137 in exon 8 of *STAT5A *showed the highest significant association with both fertilization rate and embryonic survival at the blastocyst stage. These results probably rule out the possibility of our results being due to SNPs in linkage disequilibrium with the causative mutation in the examined region although SNP153137 does not change amino acids in STAT5A protein. The survival rate and fertilization rate are clearly two different traits with a correlation of 0.15 in our ovary population, which suggests that *STAT5A *acts in two different mechanisms leading to the observed phenotypes. It is worth noting that expression of *STAT5A *reported in oocytes and in early embryonic development [8] supports our findings on the associations of this gene with fertilization and embryo survival. However, our results were obtained from in vitro experiments, which do not warrant similar results in *in *vivo experiments.

### Monoallelic Expression of STAT5A

Although many factors are involved in the early death of embryos, comparison of expression patterns between degenerative embryos and blastocysts would shed some light on the mechanisms leading to death or survival. Indeed, the occurrence of monoallelic expression of *STAT5A *was significantly higher in degenerative embryos than in blastocysts. Moreover, although a small proportion of the blastocysts that showed monoallelic expression revealed paternal expression (four out of 31), there was a clear parent-of-origin-specific trend in expression of *STAT5A*. In contrast to the monoallelic expression observed at the blastocyst stage, expression analysis of *STAT5A *in 16 different organs – obtained from fetuses at different developmental stages – revealed biallelic expression. These results are consistent with the observations of Deltour and colleagues [12] who reported biallelic expression of the *Insulin 2 *gene at Day 12.5 in the mouse yolk sac and a complete monoallelic paternal expression at Day 14.5 of development. Moreover, it is evident that parent-of-origin specific, monoallelically-expressed genes (imprinted genes) have roles in growth and embryo development, fertility, and embryonic lethality [13-15]. Thus, we conclude that STAT5A influences early embryonic survival in a developmental-stage-specific and parent-of-origin manner.

It has been shown that disruption of *Stat5 *leads to infertility in female mice as they have small-sized or absent corpora lutea, which in turn leads to significant consequences for the establishment of pregnancy [16]. Thus, our results on the effects of *STAT5A *on embryonic survival are consistent with the reported role of this gene in mouse fertility.

## Conclusion

In this study, we confirm our earlier finding that *STAT5A *is associated with embryonic survival and fertilization rate. We also show that specifically maternal monoallelic expression of this gene is associated with embryonic survival. The combination of the IVF population that was created to map genes involved in fertility traits with the strategy of choosing a candidate gene based on its role in a candidate pathway have allowed the identification of SNP153137 as a candidate SNP affecting embryonic survival and fertilization rate. This result was supported by the differential parent-of-origin expression of *STAT5A *in degenerative embryos compared to blastocysts. However, we can not exclude the possibility that SNP153137 is in linkage disequilibrium with other functional SNP(s) in *STAT5A*. Indeed, it has been proven challenging to identify causative mutations in livestock species and the number of functional mutations identified is very small probably due to the limitations of constriction of transgenic animal models in these species [17].

Given that *STAT5A *was found to be highly conserved from zebrafish to humans implies that the effects on embryonic survival and fertilization rate found in cattle could also be found in other livestock species and in humans as well. The identification of fertility genes through comparative genomics across species has been well documented in the literature as in Bonilla and Xu [18] who reported the identification of 58 genes with highly conserved male fertility function from fly to humans. These results suggest *STAT5A *as a candidate gene affecting embryonic survival and other fertility traits in humans and livestock species.

## Methods

### Assessment of survival and fertilization rates

Ovaries from 440 mature Holstein cows were collected from a local abattoir and immediately used in the IVF experiments. Fertilization of oocytes was as previously described [7,11]. In brief, oocytes were aspirated from antral follicles (> 2–6 mm), processed in different media and in incubated in maturation medium for 20–24 hours. On Day 2, oocytes were fertilized with frozen-thawed percoll-separated bull semen that had been adjusted to a final concentration of 1 million sperm/ml. Oocytes and sperm were co-incubated for a period of 18–24 h. After the fertilization period, putative zygotes were stripped of their cumulus cells by vortexing for 3 minutes, then washed 3 times in TALP-Hepes. Gametes from a total of 440 cows and eight bulls were used in the IVF experiment. Fertilization rate was calculated as proportion of cleaved embryos 48 h post fertilization out of total number of oocytes exposed to sperm. Survival rate of embryos was calculated as the number of blastocysts on Day 7 of development out of the number of total embryos cultured. Viability of blastocysts was determined as a function of the embryo's ability to attain the morphological stage of blastocyst on Day 7 of development. Embryos that failed to show cellular compaction (morula stage) on day 5 or 6 were considered non viable. Therefore only embryos exhibiting adequate compaction followed by the formation of a blastocoele on Day 7 were considered viable. Embryos were preserved in RNALater RNA Stabilization reagent (Qiagen, Valencia, CA) to avoid RNA degradation.

### Polymorphism identification and genotyping

Respect to [7], we extended our search for SNPs to include 8,998 bp upstream of *STAT5A*, all exons of *STAT3*, and 3,699 bp upstream sequences of *STAT3*. Table 1 shows only primers with which SNPs were identified. Genomic DNA was extracted from ovaries by grinding 30–100 mg from each ovary using the AquaPure Genomic DNA kit (Bio-Rad, Hercules, CA). The DNA concentration was measured using a spectrophotometer (Ultraspec 2100; Amersham Biosciences). DNA pools were constructed from 50 different ovary samples to contain 50 ng of DNA from each sample and amplified with the primers listed in Table 1. Amplification was performed in a 25-μl reaction volume, which included 50 ng genomic DNA, 50 ng each primer, 200 μM each dNTP, 2.5 μl 10× PCR buffer (Promega, Madison, WI), and 0.5 u Taq DNA polymerase (Promega). The temperature cycles were as follows: 95°C for 5 min, followed by 32 cycles of 94°C for 45 s, touchdown annealing from 63–50°C for 45 s (-2°C/cycle), 72°C for 45 s, and a final extension at 72°C for 8 min. The PCR products of the pooled DNA samples were sequenced using BigDye terminator (Applied Biosystems, Foster City, CA), and SNPs were identified by visually inspecting sequence traces. Individual cows and bulls were genotyped by sequencing.

### Embryo genotyping

Genomic DNA and RNA were extracted from embryos using Ambion kit (Applied Biosystems). Embryos were genotyped for SNP153137 (G/C) in exon 8 of *STAT5A *using primers STATF1 and STATR1 and for SNP177338 in exon 12 of *STAT3 *using primers STAT3F12 and STAT3R12 (Table 1). Amplification was performed in a 25 μl reaction volume, which included 3 μl of embryo DNA, 50 ng each primer, 200 μM each dNTP, 5.0 μl 5× PCR buffer, and 1.5 u Taq DNA polymerase (Promega). The temperature cycles were as follows: 95°C for 5 min, followed by 32 cycles of 94°C for 45 s, touchdown annealing from 65–53°C for 45 s, 72°C for 45 s, and a final extension at 72°C for 7 min. The PCR products were amplified in a nested PCR reaction using primers STAT14 and STAT13 for SNP153137 and primers STAT3F12A and STAT3R12A for SNP177338 (Table 1). The nested PCR reaction included 1 μl PCR product, 50 ng each primer, 200 μM each dNTP, 5.0 μl 5× PCR buffer, and 1.5 u Taq DNA polymerase (Promega). The temperature cycles were as described for the first PCR except the total number of cycles was set to 16. Products of the nested PCR were genotyped by digestion with the restriction enzyme B*stE*II, which allows one to distinguish alleles C and G of SNP153137. For SNP177338, PCR products were digested with the restriction enzyme M*spA1*I which allows one to distinguish alleles G and A.

### Expression analysis of STAT5A and STAT3

To analyze the expression patterns of *STAT5A *and *STAT3*, SNPs identified in heterozygous individuals were employed to distinguish between monoallelic and biallelic expression. Dams and sires of heterozygous embryos were genotyped in order to determine parental origin of monoallelically-expressed alleles. Primers were designed to amplify fragments spanning more than one exon to exclude the possibility of mistyping due to genomic DNA contamination in the RT-PCR reactions. Primers STAT14 and STAT11 were designed in exons 8 and 11, respectively to amplify a 360 bp fragment which includes SNP153137 from the *STAT5A *cDNA (Table 1). Primers STAT3-1 and STAT3-2 were designed in exons 13 and 9, respectively to amplify a 253 bp of cDNA fragment of *STAT3 *which includes SNP177338. Primers b-actin F/b-actin R (Table 1) were used to amplify 191 bp from the housekeeping gene b-actin (GenBank accession number NM_173979) cDNA as a positive control.

In order to test monoallelic versus biallelic expression in fetal tissues, organs from five fetuses at ages 68 to 90 days of age were obtained from a local slaughterhouse. All specimens were preserved in RNALater RNA Stabilization reagent (Qiagen) to avoid RNA degradation. Organs were ground with a mortar and pestle in liquid nitrogen into a fine powder, which then was used for either RNA or DNA extraction. For fetuses heterozygous for SNP153137 (n = 5), the expression pattern of *STAT5A *was analyzed in a wide range of organs: brain, ovary, liver, pituitary, adrenal gland, lung, skeletal muscle, heart, spleen, teste, cotyledon, mammary gland, rib, kidney, eye, and intestine. The RT-PCR was performed using Qiagen OneStep RT-PCR Kit (Qiagen). The RT-PCR cycling conditions included incubation at 50°C for 30 min, 95°C for 15 min, and then touchdown PCR conditions, as described for genomic DNA PCR amplifications. The RT-PCR products for SNP153137 and SNP177338 were genotyped by digestion with the restriction enzymes *BstE*II and *MspA1*I, respectively, as described for embryo genotyping.

### Statistical analysis

Differences in monoallelic expression of STAT5A and in parent-of-origin for heterozygous degenerative embryos vs. blastocysts were tested through a Pearson's Chi-squared test. Given the low number of counts in some of the cells, p-values obtained from a Monte Carlo test with 2000 replicates and those obtained through Yates' continuity correction were reported. All analyses were performed with the stat package of Rsoftware v. 2.5.1 .

Association between SNPs and fertilization and survival rate at Day 7 were analyzed using the following mixed linear model:

*y*_*ijk *_= *μ *+ *o*_*i *+ _*s*_*j *_*+ SNP*_*ijk *_*+ e*_*ijk*_

where *y*_*ijk *_represents in turn, the survival or fertilization rate of a batch of ova *k *from ovary *i *fertilized with semen from sire *j*; *μ *represents a general constant (mean) for the trait considered; *o*_*i *_represents the random effect of the individual ovary from which ova were harvested; *s*_* j*_ represents the random effect of sire used in the fertilization; *SNP*_*ijk *_represents the fixed effect of the genotype for the SNP considered; and *e*_*ijk *_represent the residuals, assumed normal and independent with mean 0 and variance **I***σ*^2^_*e*_. Ovaries and sires were both assumed uncorrelated in the analysis, with variance structures **I***σ*^2^_*o *_and **I***σ*^2^_*s *_respectively. After data editing, ovaries from which fewer than 4 eggs were harvested were excluded from the analysis. All analyses were performed with the function *lmer *of the *lme4 *package of R software v. 2.5.1 .

## Authors' contributions

HK designed the study, performed expression analysis, and wrote the manuscript. CM performed the statistical analysis. RLM and JJR were responsible for ovaries and semen collection, fertilizations, and collecting survival and fertilization data. VS performed DNA extraction and genotyping.

## Supplementary Material

Additional file 1 **Supplemental table one**. SNP numbers and locations presented in Figure 2. Click here for file
