# Supplementary material for: Monoallelic maternal expression of STAT5A affects embryonic survival in cattle
Source: BMC Genet. 2009 Mar 10;10:13. doi: 10.1186/1471-2156-10-13 (PMC2662876; doi:10.1186/1471-2156-10-13)
Supplement: Additional file 1 — Supplemental table one. SNP numbers and locations presented in Figure 2. [file 1471-2156-10-13-S1.doc]

**Supplementary Table 1: SNP numbers and locations presented in Figure 2.**

| **SNP** | **Nucleotide number** | **location** |
| --- | --- | --- |
| 1 | 134,357 | Upstream STAT5A |
| 2 | 134,828 | Upstream STAT5A |
| 3 | 134,920 | Upstream STAT5A |
| 4 | 135,162 | Upstream STAT5A |
| 5 | 135,249 | Upstream STAT5A |
| 6 | 135,397 | Upstream STAT5A |
| 7 | 137,887 | Upstream STAT5A |
| 8 | 138,012 | Upstream STAT5A |
| 9 | 138,242 | Upstream STAT5A |
| 10 | 138,299 | Upstream STAT5A |
| 11 | 138,337 | Upstream STAT5A |
| 12 | 138,338 | Upstream STAT5A |
| 13 | 138,596 | Upstream STAT5A |
| 14 | 138,653 | Upstream STAT5A |
| 15 | 144,059 | Intron 4, STAT5A |
| 16 | 144,361 | Intron 4, STAT5A |
| 17 | 153,137 | Exon 8, STAT5A |
| 18 | 153,827 | Intron 9, STAT5A |
| 19 | 153,866 | Intron 9, STAT5A |
| 20 | 154,186 | Intron 9, STAT5A |
| 21 | 154,261 | Intron 9, STAT5A |
| 22 | 154,458 | Intron 9, STAT5A |
| 23 | 154,596 | Intron 9, STAT5A |
| 24 | 154,598 | Intron 9, STAT5A |
| 25 | 155,159 | Intron 9, STAT5A |
| 26 | 156,483 | Intron 12, STAT5A |
| 27 | 171,005 | Intron 19, STAT3 |
| 28 | 177,338 | Exon 12, STAT3 |
| 29 | 197,390 | Upstream STAT3 |
| 30 | 197,429 | Upstream STAT3 |
| 31 | 197,456 | Upstream STAT3 |
| 32 | 197,558 | Upstream STAT3 |
| 33 | 197,602 | Upstream STAT3 |
| 34 | 197,608 | Upstream STAT3 |
| 35 | 197,713 | Upstream STAT3 |
| 36 | 197,718 | Upstream STAT3 |
| 37 | 197,740 | Upstream STAT3 |
